# Dupilumab Reduces Pruritus in Twins With Sjögren–Larsson Syndrome

**DOI:** 10.1111/pde.70073

**Published:** 2025-10-05

**Authors:** Kennedy Gallagher, Rame Yousif, Kathryn Hummel, Amy S. Paller

**Affiliations:** ^1^ Department of Dermatology Northwestern University Feinberg School of Medicine Chicago Illinois USA; ^2^ Department of Pediatrics Northwestern University Feinberg School of Medicine Chicago Illinois USA; ^3^ Division of Pediatric Dermatology Ann and Robert H. Lurie Children's Hospital of Chicago Chicago Illinois USA

**Keywords:** congenital ichthyosis, dupilumab, pruritus, Sjögren–Larsson syndrome

## Abstract

Sjögren–Larsson Syndrome (SLS), now termed *ALDH3A2*‐syndromic epidermal differentiation disorder (sEDD), is a rare genetic disorder marked by thickened skin, spasticity, and intellectual disability. Intractable pruritus is a nearly universal and debilitating feature of SLS that remains poorly managed by current therapies. We describe 4‐year‐old twin girls with genetically confirmed SLS who showed significant and lasting improvement in itch following treatment with dupilumab, a biologic targeting interleukin‐4 receptor signaling. Within 6 months, pruritus severity scores declined markedly, improving sleep and quality of life without adverse effects, supporting further investigation of dupilumab for SLS‐associated itch.

## 1 Introduction

Sjögren–Larsson syndrome (SLS; new nomenclature *ALDH3A2*‐syndromic epidermal differentiation disorders (sEDD) [1]) is a rare autosomal recessive disorder caused by pathogenic variants in *ALDH3A2*, which encodes for the enzyme fatty aldehyde dehydrogenase (FALDH). FALDH deficiency disrupts normal fatty acid metabolism, leading to pathologic accumulation of fatty alcohols and aldehydes, particularly within the lipid membranes of the skin and central nervous system [2]. Clinically, SLS is characterized by the classical triad of marked thickening and yellow discoloration of the skin, spastic diplegia or quadriplegia, and intellectual disability. Intractable pruritus is a nearly uniform characteristic, further complicating quality of life for affected infants and children. Treatment for SLS is supportive and aimed at alleviating neurologic symptoms (e.g., spasticity, seizures) and cutaneous manifestations [2].

Dupilumab, a biologic that targets the interleukin (IL)‐4 receptor (IL‐4R), is currently approved for treating patients 6 months of age and above with moderate‐to‐severe atopic dermatitis. Signaling through the IL‐4R augments itch responses through a variety of receptors on dorsal root ganglia, including the IL‐31 receptor, and may have direct stimulatory effects on non‐histaminergic itch [3, 4]. We describe a set of twins with SLS/*ALDH3A2*‐sEDD who experienced dramatic reduction in associated pruritus with dupilumab treatment, maintained for at least a year after initiation.

## 2 Case Report

**FIGURE 1:**
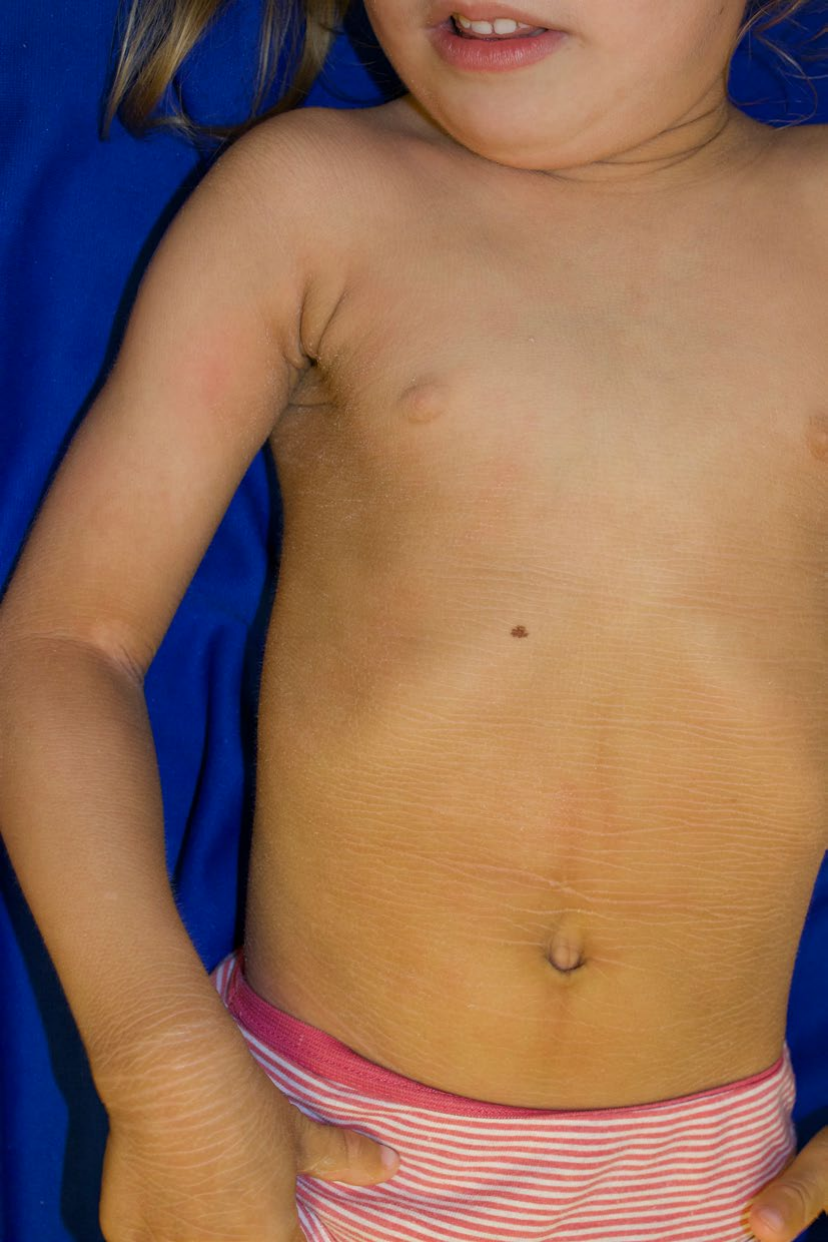
Yellow‐hued, thickened skin of Sjögren–Larsson syndrome. The typical accentuation of skin lines and focal area of desquamation at the antecubital area, revealing underlying erythema.

Two 4‐year‐old female twins, recently relocated to the United States from Ukraine, presented for evaluation of marked skin thickening and intractable pruritus. Both were born prematurely at 34 weeks gestation and had erythroderma at birth without a collodion membrane. Their medical histories were notable for global developmental delays, including significant motor, speech, and growth impairment, as well as spastic diplegia and several ophthalmologic issues, among them esotropia, blepharitis with chalazia, and amblyopia (both twins); myopia and astigmatism (Twin A); and severe photophobia (Twin B). Additional comorbidities included scoliosis in Twin A, and intraventricular hemorrhage at birth with hydrocephalus requiring shunt placement, seizures, and dental caries in Twin B. The family denied a history of skin disease or known consanguinity. Skin examination was typical for SLS/*ALDH3A2*‐sEDD [1] with a yellowish skin tone, generalized skin thickening with marked accentuation of skin lines, particularly at flexural areas, and focal areas of desquamation (Figure 1). The family reported intense pruritus, with an average 7‐day itch Numerical Rating Scale (NRS) of 7/10 and peak itch of 10/10 for both twins, significantly disrupting sleep. Treatment was limited to full‐body emollient application up to five times daily, although 40% urea cream and calcipotriene 0.005% ointment were tried and poorly tolerated. Trials of triamcinolone 0.1% ointment, tacrolimus 0.03% ointment, and tazarotene 0.1% cream minimally reduced the itch and skin thickening. Genetic testing confirmed the diagnosis with compound heterozygosity for pathogenic variants in *ALDH3A2* (c. 1157A>G and c.734A>C).

Both twins were initiated on dupilumab 300 mg every 4 weeks. After 3 months, the family reported substantial improvement in sleep, and both average and peak itch NRS decreased to 6/10. By 6 months, baseline immunoglobulin E (IgE) levels, initially within the normal range (< 108 kU/L; Twin A: 11.0, Twin B: < 2.0), had declined further (Twin A: 4.34, Twin B: < 2.0). By 16 weeks after initiation, pruritus improved to 2–3/10 average and 5 for 7‐day peak pruritus, maintained at 1 year on medication. The key anti‐pruritic impact of dupilumab was confirmed by peak scores of 8–9/10 during a 3‐month hiatus off drug and increases of itch (peak 7–8/10) during the week prior to each every 4‐week injection, prompting a switch to every 3 weeks dosing. No treatment‐related adverse events have been observed to date.

## 3 Discussion

Administration of dupilumab, a biologic indicated for atopic dermatitis, markedly reduced intractable itch and improved sleep quality in twin sisters with SLS/*ALDH3A2*‐sEDD, suggesting a promising therapeutic approach for this life‐altering disease. In SLS, pruritus is persistent, poorly responsive to standard topical treatments, and is a major contributor to morbidity and reduced quality of life. Despite being a defining feature of SLS, itch has received limited attention in both the clinical literature and therapeutic management. The pathogenesis of pruritus in SLS remains incompletely understood but is likely multifactorial, involving the accumulation of fatty alcohols and aldehydes that lead to structural abnormalities of the skin and increased transepidermal water loss [2]. Disruption of skin barrier integrity may, in turn, contribute to peripheral nerve dysfunction or activation of non‐histaminergic pruriceptive pathways, including those mediated by IL‐4 and IL‐31, which are key cytokine drivers of pruritus in atopic dermatitis and other inflammatory dermatoses.

Alternative systemic therapies targeting pruritus, such as nemolizumab, an IL‐31 receptor antagonist, and Janus kinase inhibitors, which suppress downstream Janus kinase/signal transducers and activators of transcription (JAK–STAT) signaling, are currently limited to adolescents and adults. In contrast, dupilumab was chosen based on its excellent safety profile, including in pediatric populations, and the lack of need for laboratory monitoring. By blocking the IL‐4Rα subunit and thus the IL‐4R/IL‐31R heterodimer activated by IL‐4 and IL‐13, dupilumab reduces Type 2 inflammation but also suppresses downstream pruritus signaling within the dorsal root ganglia. Its antipruritic efficacy is evidenced by its on‐label use for prurigo nodularis, in which IL‐31 induced itch is central to disease pathogenesis. Dupilumab has also shown value for numerous off‐label indications, among them genetic skin disorders, such as epidermolysis bullosa pruriginosa, a genetic blistering disorder also characterized by intractable pruritus, and Netherton syndrome (*SPINK5*‐sEDD [1]), both disorders with elevated serum IgE levels [5, 6, 7, 8, 9]. In contrast, the twins in this report had normal IgE levels at baseline, suggesting that the benefits of dupilumab in SLS may occur through mechanisms affecting itch unrelated to Type 2 immune activation or IgE. Given the remarkable response of SLS‐associated pruritus to dupilumab in this case, formal investigation of this and other targeted biologics is warranted to establish evidence‐based treatments for this underserved and profoundly impacted patient population.

## Conflicts of Interest

A.S.P. has been an Investigator for AbbVie, Biomendics, Dermavant, Eli Lilly, Incyte, Johnson & Johnson Innovative Medicine, Regeneron, UCB; a Consultant for: Abeona, Arcutis, BioCryst, Boehringer‐Ingelheim, Castle Creek, Chiesi, Dermavant, Johnson & Johnson Innovative Medicine, Krystal, LEO, Lilly, L'Oreal, MoonLake Immunotherapeutics, Peltheos, Quoin, Regeneron, and Sanofi; and on the Data safety monitoring board for AbbVie, Abeona, BioCryst, Daiichi Sankyo, and Galderma. The other authors have no conflicts of interest.

## Data Availability

Data sharing not applicable to this article as no datasets were generated or analyzed during the current study.
